# Mental health and diabetes self-management: assessing stakeholder perspectives from health centers in Northern Mexico

**DOI:** 10.1186/s12913-021-06168-y

**Published:** 2021-02-25

**Authors:** Benjamín Aceves, Manuel Ruiz, Maia Ingram, Catalina Denman, David O. Garcia, Purnima Madhivanan, Cecilia Rosales

**Affiliations:** 1 Department of Health Promotion Sciences, Mel and Enid Zuckerman College of Public Health, University of Arizona, 1295 N Martin, Tucson, AZ 85724 USA; 2 Division of Public Health Practice and Translational Research, Mel and Enid Zuckerman College of Public Health, University of Arizona, Phoenix, AZ USA; 3 Social Interventions Research and Evaluation Network, University of California,San Francisco, San Francisco, CA USA; 4 Center for Health and Society Studies, El Colegio de Sonora, Hermosillo, Sonora Mexico; 5 Department of Medicine College of Medicine, University of Arizona, Tucson, USA; 6 Public Health Research Institute of India, Mysore, India

**Keywords:** Mental health, Diabetes, Mexico, Health services

## Abstract

**Background:**

People living with diabetes have an increased risk of developing mental health issues. Mexico has observed a high prevalence of people living with diabetes suffering from mental health issues, such as anxiety and depression. Self-management programs have demonstrated promise in helping participants address and prevent not only physiological health complications but mental health issues as well. This qualitative study aimed to understand the mental health benefits of a diabetes self-management intervention for health centers in Northern Mexico and opportunities for improvement through assessing stakeholder perspectives.

**Methods:**

Trained research staff used a semi-structured questionnaire guide to conduct all interviews and focus groups from February–May 2018. Individual interviews (*n* = 16) were conducted face-to-face at four health center sites among all health center directors and key staff located throughout the state of Sonora. One focus group (*n* = 41) was conducted at each of the four health centers among intervention participants. Directed content analysis was used to establish themes by understanding relationships, identifying similar experiences, and determining patterns across datasets.

**Results:**

In total 57 health center directors, health center staff, and intervention participants were involved in the interviews and focus groups across the four health centers. Overall the analysis identified four themes throughout the data, two were categorized as benefits and two as improvements. The primary themes for participant benefits were an increase in self-efficacy and social support to manage their chronic conditions. These were evident from not only participant perspectives, but health staff observations. Conversely, increased family involvement, and increased mental health integration and services within diabetes care were identified themes for opportunities to improve the intervention to be more inclusive and holistic.

**Conclusion:**

All stakeholders observed the benefits for intervention participants and opportunities for more inclusivity of the family and integration as well as an increase in mental health services. The themes identified demonstrated a need to more proactively enhance and utilize diabetes self-management as a means to improve mental health outcomes among people living with diabetes in Mexico. This is an opportunity to employ a more comprehensive approach to diabetes self-management, and integrate mental health services into overall diabetes care.

**Trial registration:**

www.ClinicalTrials.gov, identifier: NCT02804698. Registered on June 17, 2016.

## Background

Worldwide, people living with diabetes are 1.2 times more likely to suffer from anxiety disorders and 1.4 times more likely to suffer from depression than those without chronic disease [1]. This is particularly worrisome for Mexico, given Type-II diabetes mellitus in the country has increased over several years from a prevalence of 9.2% in 2012 to 10.3% in 2018 [2, 3]. In 2019, the Mexican Health and Aging Study found that individuals living with diabetes were more likely to experience severe depressive symptoms and issues sleeping [4]. Similarly, among 820 Mexican outpatients living with diabetes, investigators found a prevalence of depression to be 48.3%, while the anxiety disorder rate was 55.1% [5]. Diabetes distress and depression are associated with a high risk of mortality, poor disease management, chronic disease related complications, and poor quality of life [6]. Low-income individuals living with diabetes in low- and middle-income countries are more likely to suffer from depression and other mental health issues, and in effect can lead to a decrease in quality of life [7–9]. Mexico’s low-resource setting makes it even more challenging to obtain clinical resources and adequate quality of care, as well as to engage individuals living with diabetes into patient education programs [10].

Mexico’s Ministry of Health provides a range of health care services to low-income citizens with diabetes through health centers located throughout the country [11]. These services range depending on the location, but much of the time include screening for diabetes, diagnosis, self-management programs, mental health services, and medication at no cost to the individual [11]. The availability of these health services also ranges across health centers given the range of bureaucratic and institutional barriers, as well as presidential transitions of power [12]. *Grupos de Ayuda Mutua* (GAMs), or support groups for people living with diabetes, have been designated as a self-management resource to help individuals within health centers, however, currently it lacks an evidence-based program curriculum to guide sessions [13, 14]. The World Health Organization has long recognized a need for evidence-based guidelines within national diabetes self-management programs to ensure a reduction in diabetes-related complications [15, 16].

Independently, current mental health services for people in Mexico lack infrastructure and trained professionals to vastly implement programs or disseminate resources throughout the republic [17]. The majority of existing mental health services are centered within very large cities, while less populous areas will have limited professional or student mental health providers [17]. Given these resource-limited and segmented services, the need to fulfill several health needs among people living with diabetes is apparent—specifically related to self-management and mental health services.

A binational research team in Sonora, Mexico collaborated with the Ministry of Health to fill the void of comprehensive diabetes self-management program through the implementation of *Meta Salud* Diabetes (MSD) [18]. MSD is a diabetes self-management intervention consisting of 13 weekly sessions led by health staff (i.e.-nurses, doctors, and community health workers), in which participants learn and discuss topics related to diabetes complications, mental health, health services, and lifestyle changes (including nutritional and physical activity habits) [18]. Diabetes self-management and education programs have been long proven effective in significantly reducing depressive symptoms and increasing protective factors, such as strong social support [19, 20]. MSD builds upon the evidence on self-management through the utilization of the salutogenic model, which applies health promotion strategies through addressing mental health indicators—such as motivation and distress [18]. The specific health promotion strategies used to target mental health issue included:

1. Building knowledge surrounding the relationships between physiological and psychological health status
2. Empowering participants on practical methods and existing resources available for managing their chronic disease
3. Creating an open space to facilitate dialogue on the issues and struggles people face related to diabetes
4. Establishing and promoting a holistic approach to self-management through discussions and interactive activities that address several social determinants of health

In targeting mental health indicators, the intervention aims to increase adherence to self-management and decrease distress, which in the long-term would decrease the risk of diabetes-related complications [18]. The intervention has already demonstrated significant improvements in mental health outcomes during the hybrid implementation-effectiveness trial [21]. Measurements taken, using the Spanish version of the Problem Areas in Diabetes Scale, indicated significant improvements in diabetes distress [21, 22]. The objective of this qualitative study was to assess the perspectives of health center directors, staff, and intervention participants on the perceived mental health benefits of the MSD intervention, as well as any related opportunities for improvement. In addition, health center staff and directors’ perspectives on current mental health services provided to people living with diabetes were accessed.

## Methods

### Setting

The study was conducted in the state of Sonora, located in the northwest region of Mexico, in collaboration with the Ministry of Health. MSD participants, along with health center staff and directors, were recruited from four selected health center sites, which completed the original MSD implementation effectiveness trial and agreed to participate in a qualitative study. The opportunity sample of health centers was also chosen based on distinct locations in the North, West, Central, and South regions of the state, which was meant to yield a diverse geographical richness to the qualitative data. Health centers located in the North and West regions of the state were located in cities with populations less than 100,000, while health centers in the Central and South regions were in major cities with populations above 400,000. All health centers would be considered primary care facilities, which include services such as health promotion and disease prevention programs, diagnosis and treatment of chronic and acute illnesses, referral programs, while typically staffing one or less designated mental health professional.

Key stakeholder interviews and focus groups were conducted by experienced female university researchers (Authors: MI [MPH]; CD [PhD]; CR [MD, MPH]) who were either involved in the implementation of the MSD intervention and/or providing services to diabetes enrollees. It is important to note researchers’ identity, given how gender norms influence workplace environments in Mexico. Researchers had established relationships with health directors, staff, and intervention participants, which had been fostered over several decades of conducting research within the state.

Focus groups were conducted with the following inclusion criteria: people, over 18 years of age, living with diabetes receiving services from one of four health centers, and who completed the MSD intervention within the past 6 months. The focus groups were conducted with one of the researchers, with a research assistant to take observational notes. Twelve health center staff (3 per site) and all four center directors participated in the interviews, equally representing all regions. In total 41 MSD participants were involved in the one-session focus groups, the attendance was as follows: North (8), West (11), Central (13), South (9).

The study received human subjects’ approval from the University of Arizona Institutional Review Board. All participants provided written consent after being informed about the research process and study topics. Participation in the study was completely voluntary with no impact on employment or future health services, in addition, all identifiable information was kept confidential. All of the audio recordings and transcriptions were kept in a secure electronic database, without any identifiable information. Participants were aware that researchers would utilize this information to improve health services and the intervention, indicative of previous work conducted in the region.

### Data collection and analysis

Trained research team members used a semi-structured questionnaire guide to conduct all interviews and focus groups in Spanish from February–May 2018 within health centers in private rooms. Participants were asked to expand upon responses or share additional information. Guides were constructed considering social-ecological contextual factors within the health centers influencing MSD participants’ experience, similar to dynamics and influences that are described and provided in previous published work [23]. Interviews and focus groups were audio-recorded and transcribed verbatim, and observational field notes were taken to give transcriptions behavioral context during analysis. Both transcriptions and observational field notes were completed by qualitative research staff, who did not participate in facilitating interviews or focus groups, nor a part of the analysis. Interviews were designed to last 60 min and focus groups approximately 80 min.

#### Methodological rigor

**Fig. 1:**
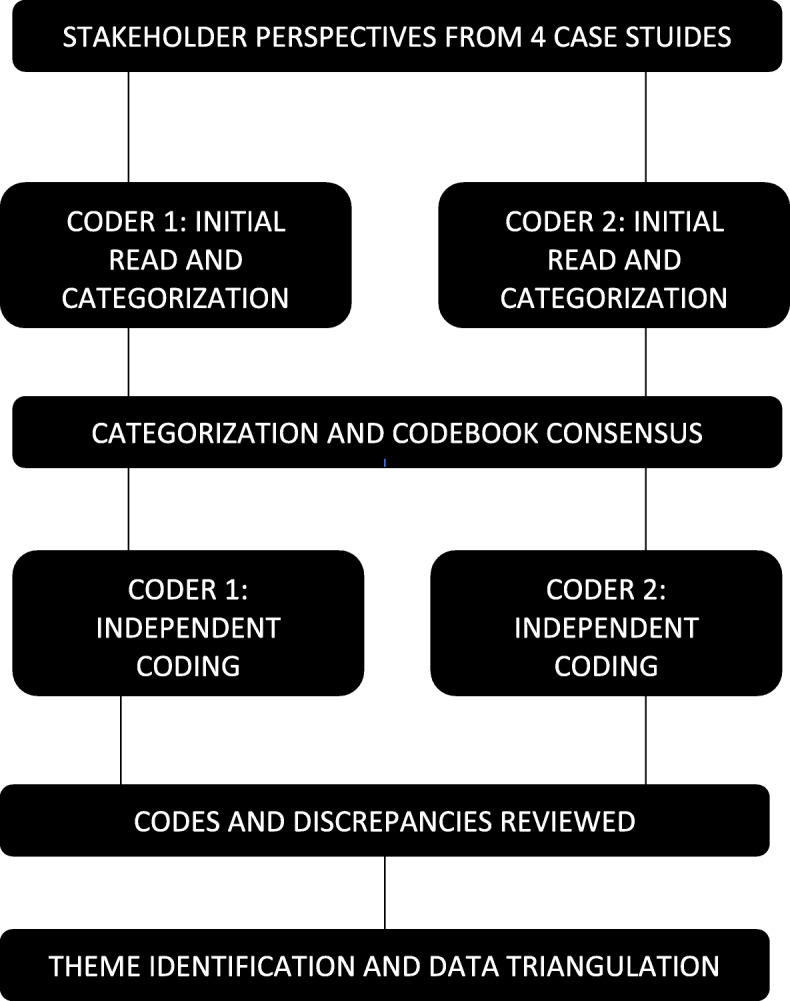
Analytical Approach

All data were analyzed in Spanish by two bilingual coders (BA, MR), subsequently, exerts were translated when needed for reporting of results. Directed content analysis was used to code and extract themes from the data using NVivo 12 [24]. This analytical approach was utilized given our initial effectiveness trial quantitative demonstrated mental health benefits, but this study was to provide additional contexts and descriptions on the benefits, health services, and opportunities for improvement. As seen in Fig. 1, the two independent bilingual coders initially read all transcriptions and began initial categorizing data along identification of benefits and improvements. Data categorization was reviewed and discussed, then subsequently coders developed an agreed codebook based on recurrent topics. All data was then coded independently by coders. Discrepancies in coding were discussed among both coders until consensus was reached. The themes were created in an iterative process through the identification of similar experiences and salient patterns. In addition, the three distinct stakeholder perspectives were utilized to triangulate specific health center contexts, services, and patient experiences.

## Results

Overall 57 stakeholders’ perspectives were assessed for this qualitative study. Twelve health center staff and all four center directors participated in the interviews lasting approximately 60 min, equally representing all regions. The focus groups conducted had a duration between 79 and 85 min. As seen in Table 1, the majority of participants were female (63%), and the largest number of participants were from the Central region health center, followed by West, South, and North. Four emergent themes were identified across the three stakeholder groupings with two overarching categorizations. Two of the themes were identified as benefits, increases in self-efficacy and social support. In contrast, there were two opportunities for improvement specifically concerning increased family involvement, and increased mental health integration and services within diabetes care.

**Table 1:** Study Participants (*n* = 57)

Characteristic	Number of Participants (%)
***Total Participants***	
Health Center Directors	4 (7)
Medical Doctors	4 (7)
Nurses	6 (11)
Community Health Workers	2 (4)
MSD Participants	41 (71)
***Sex***	
Male	21 (37)
Female	36 (63)
***Region***	
North	12 (21)
West	15 (26)
Central	17 (30)
South	13 (23)

### Identified benefits

#### Increased self-efficacy

MSD participants consistently emphasized the increased self-efficacy gained from the intervention. This was expressed through increased capability for managing their chronic conditions, but also in actively working against the common negative social stigmas of diabetes. Many participants felt an increased capability to take how control of their health status, and an ability to actively work to improve it.

*“People tell us, ‘Poor you! You are a diabetic.’, right everyone? This is true diabetics have several health issues, but now I can control these issues and know how to move forward and am motivated to improve, the intervention has helped me move forward in my self-management.”*

*-MSD Participant, female*

Participants felt an increase in self-efficacy to manage their diabetes through a better understanding of the specific behaviors and strategies needed for self-management. These learned behaviors and strategies were identified as having interconnected impacts on their psychological and physiological health.

*“I have learned that I need to eat small portions and exercise, and well emotions, I have learned to try to manage my emotions. But they are all connected, and I have learned how to do all of this. Now, I am doing this, now I know how to care for myself.”*

*-MSD Participant, female*

Health center staff also observed the increase in self-efficacy through the motivation MSD participants demonstrated during the intervention. As a result, an improvement in the mental health state of several participants was observed.

*“This really changed their lives. Many of them either didn’t accept their diabetes or were depressed. However, in the 13 weeks they changed and are no longer depressed, they learned what they needed to change and they did it, they were able to make the changes.”*

*-Community Health Worker, female*

#### Increased social support

The support group structure of the MSD intervention and the interactive dialogues facilitated through sessions assisted in establishing social bonds between participants. The social bonds grew outside of the clinic setting and continued after official GAM meetings.

*“…what they found in the group was a comradery. After finishing Meta Salud Diabetes, they found a family and lots of support, in fact, they leave the meetings at 11 and after, go to someone’s house and they socialize.”*

*-Nurse, female*

Participants also noted that the MSD intervention helped facilitate improvement in their ability to connect with others with diabetes, which in turn helped them keep on track with their self-management. This social support transformed many participants’ motivation.

*“We all hang out, we are like friends, we are like a family, a family that supports one another and can motivate one another.”*

*-MSD Participant, female*

Growth in social support during the intervention implementation was a driver for alleviating depressive symptoms among many GAM participants. It assisted people in expressing their emotions and creating a dialogue concerning issues encountered.

*“I have seen that being in the GAM group (now) helps a lot, it helps people who arrive sad, depressed, somber, not wanting to speak. And the same people now seem very motivated and keep coming. Speaking with people helps relax them. In fact, my friend here, when she arrived she looked very serious and would not speak, but now little by little she is opening up and keeps coming.”*

*-MSD Participant, male*

### Opportunities for improvement

#### Increased family involvement

The need to integrate family members into diabetic care, particularly mental health, was apparent throughout participant focus groups. There was an evident need to involve family members in their self-management to explain not only the physiological but psychological changes people may experience related to their chronic disease.

*“Yes, the problem is that sometimes the physical and emotional changes of people with diabetes is very apparent, but the family sometimes does not understand. It is not that I don’t like meat, I just cannot eat red meat. They do not know that I do not have balanced sugar levels, and that cells in my body are not able to absorb sugars because of things like meat. As Gabriela said, sometimes I am not in the mood, I do not have the motivation, it’s the depression in diabetics, it’s very incomprehensible for families.”*

*-MSD Participant, male*

Participants recognized the MSD intervention as an opportunity to integrate family members into their diabetes care and support them in establishing healthy behavioral habits.

*“I would like to see in the program, I don’t know, a meeting with our families where we talk about the importance of supporting diabetics. Maybe it would a meeting where we invite family members to familiarize themselves with the importance of supporting people because that is what we were taught, that our family should support our diet and physical activities. These are people that are sick and need a treatment, but the family does not see it that way.”*

*-MSD Participant, female*

#### Increased mental health integration and services within diabetes care

Health center leadership was aware of the need to address mental health issues among their people living with diabetes and the contribution of MSD to participant well-being. One director explained how the MSD intervention assisted staff and GAM participants in specifically integrating mental health and diabetes control, but the need to do more.

*“We have detected many sick people, that are alone, and are suffering, and that’s why I consider that now GAM helps people, it helps with adherence to treatment, which is very important, no? I also believe there should be more integration of diabetic patients into behavioral services, no? Psychiatry, or more, I just think there are more determinants of health.”*

*-Health Center Director, male*

Health center personnel also stated a need to work with participants and establish strong relationships to identify mental health issues and better coordinate into diabetes care.

*“It makes them depressed, it makes them anxious…well, I just don’t go and see the degenerative chronic issue, but also I want to see that they have emotional tranquility, and little by little I want to see that they trust in me, that way we can create a good treatment that considers emotional health.”*

*-Health Center Director, male*

In addition, health center staff expressed a need to prioritize mental health services as an essential part of the primary and coordinated care provided to people living with diabetes.

*“We are a healthcare institution. So we should be providing services in all areas, staff: psychologists, dietitians, physical trainers, etc…but as I have said for a while now there is a problem with our situation, obstacles—there is little support from administrators….what we need here is to have staff, trained (health) professionals in their area of expertise…to provide care to patients consistently.”*

*-Nurse, female*

## Discussion

There were clearly mental health benefits to engaging in the MSD intervention, specifically related to self-efficacy and social support. Improved mental health among people living with diabetes increases engagement in protective behaviors, promotes healthy habits, and improves overall physiological health [19, 20]. Results from the study also identified opportunities to improve the intervention through better increased presence of family members and mental health services. Given the limited evidence surrounding diabetes self-management intervention and mental health within LMICs and Mexico [25], this study helps fill gaps in the literature on implementing diabetes self-management programs, which address mental health, within resource-limited health systems.

MSD participants and health center staff described greater self-efficacy related to diabetes management, which contributed to improved mental health particularly among those experiencing depression. Self-efficacy was described as increased capability and empowerment needed to implement healthy habits into their lifestyle. Social support was an interpersonal benefit stated by participants and health staff throughout interviews and focus groups. The social bonds created among group members addressed mental health issues among participants related to isolation due to financial constraints and provided a space to express their struggles with the chronic disease. Increased social support is a benefit identified in several diabetes self-management interventions that emphasize the role of peers in providing emotional support and dealing with depression [26–28]. The intervention through increasing self-efficacy and social support addressed potential psychological complications (related to feelings of isolation and anxiety) associated with diabetes.

While peer social support was a defined benefit, participants also reported that the MSD intervention was an opportunity to increase family members’ understanding of the experience of living with diabetes. As several participants voiced, family members have a strong influence on their health behaviors and mental health; involving them in the intervention would better ensure the prioritization of lifestyle changes in the home that support improved mental health outcomes. Family involvement is supported by the literature and indicates that it affords an additional opportunity for emotional support, as well as companionship [29]. Future MSD intervention efforts should explore opportunities to involve family members during interactive activities, as well as develop strategies to share information.

Health center staff and leadership also felt that participants benefited from the integration of mental health topics into diabetes management but felt this could have reached farther. The MSD curriculum addresses various mental health topics throughout the intervention, which assisted in promoting protective physiological and psychological health behaviors. This integration allowed staff to approach diabetes care in a holistic process that accounts for not only physiological, but psychological complications as well. While the process also facilitated the integration of mental health topics, it simultaneously identified gaps in mental health services and a need for increased access to mental healthcare professionals during the MSD implementation process. The results indicated an overall lack of mental health professionals within health centers, specifically the absence of consistent professionals involved in diabetes care. This essentially leads to an overall lack of mental health services within diabetes care. Comprehensive approaches to diabetes management that include not only mental health services, but also addressing various social determinants of health are necessary to provide adequate care to low-income populations living with diabetes. This is reflected in reviewing the literature calling for more integration of mental health services in diabetes care among LMICs, particularly among low-income populations given the perpetual cycle of disease and poverty [7, 25, 30].

### Limitations and strengths of the study

There were limitations to this qualitative study. Firstly, these findings have encountered certain bias considering the interviewers and focus group facilitators had initial and close relationships with the participants. This was addressed by attempting to match participants with interviewers and facilitators who were unfamiliar with one another. However, before interviews and focus groups, confidentiality and the ability to skip questions were noted before starting any study activity. In addition, given several external factors limited sociodemographic data was collected, however, this was in the interest of participants.

The primary strengths of the study were related to data collection from various stakeholders throughout the state and various positions within health centers, which facilitated a reach beyond the perspective of a single group. This triangulation was critical to understanding improvements necessary and feasible within the entire health system structure. This is also reflected in providing distinct perspectives from various regions of the state of Sonora. This study was also conducted in Northern Mexico, a region that is completely understudied and lacks a presence with health-related literature.

## Conclusion and future directions

As the diabetes epidemic in Mexico evolves, the health care system must incorporate comprehensive approaches that involve mental health services. Low-income people living with diabetes are a subpopulation in urgent need of mental health services, given their risk of increased stress, depression, and other psychological complications due to the vulnerability of their socio-economic status and limited treatment options [7]. MSD provides an opportunity to offer a diabetes self-management intervention to low-income individuals that promotes a comprehensive approach, inclusive of mental health topics, and promotes integrating services. As demonstrated, social efficacy and social support are central to ensure positive mental health outcomes for people living with diabetes, which was achieved within the MSD intervention. This intervention has the potential to be replicated in other LMICs, given the contextual factors faced by similar health systems that account for in MSD.

## Data Availability

Qualitative datasets contain sensitive information that could identify participants, study sites, and therefore is not publicly available. Data supporting findings are available on request from the corresponding author BA.

## Abbreviations

GAM: Grupos de Ayuda Mutua
LMICs: Low- and Middle-Income Countries
MSD: Meta Salud Diabetes
